# Evaluation of the Efficacy and Safety of Intravenous Immunoglobulin (IVIG) in Moderate-to-Severe Hospitalized COVID-19 Patients: A Randomized, Open-Label Parallel-Group Study

**DOI:** 10.1155/2024/7209380

**Published:** 2024-05-21

**Authors:** Sachin Gautam, Govind Mawari, Mradul Kumar Daga, Naresh Kumar, Harpreet Singh, Sandeep Garg, Suresh Kumar, Monika Gajendrakumar, Mahak Golani, Ishan Rohatgi, Sayan Sarkar, Shubham Kaushik, Manish Kumar Jha, Sweety Mehra

**Affiliations:** ^1^Department of General Medicine, Maulana Azad Medical College & Associated Lok Nayak Hospital, New Delhi 110002, India; ^2^Centre for Occupational and Environmental Health (COEH), Maulana Azad Medical College, New Delhi, India; ^3^Department of Internal Medicine & Infectious Diseases, Institute of liver & Biliary Sciences, Vashant Kunj, New Delhi-110070, India

## Abstract

**Purpose:**

Since February 2020, the world has been overwhelmed by the SARS-CoV-2 outbreak, and several patients suffered interstitial pneumonia and respiratory failure requiring mechanical ventilation, threatening the capability of healthcare systems to handle this amount of critical cases. Intravenous immunoglobulins (IVIG) possess potential immunomodulatory properties beneficial for COVID-19 patients, yet evidence supporting IVIG as adjunctive therapy remains sparse. This study evaluated the outcomes of adjunctive IVIG with the standard of care (SoC) in moderate-to-severe COVID-19 patients.

**Methods:**

This randomized study included 59 moderate-to-severe COVID-19 patients with known comorbidities. One arm (*n* = 33) received high-dose IVIG (400 mg/kg/day) within 48 hours for five days alongside SoC, while the other arm (*n* = 26) received SoC, comprising steroids, enoxaparin, and remdesivir. The primary endpoint was clinical improvement, as measured by the National Early Warning Score 2 (NEWS2) and discharged/death proportions. Secondary outcomes included IVIG safety, hospitalization duration, changes in oxygen saturation, inflammatory markers, IgG titer, CTSS (CT severity score), and radiological findings.

**Results:**

There was an improvement in the NEWS2 at the end of treatment in the IVIG arm (5.67 vs. 5.96). A significant absolute effect improvement (Day 1 vs. Day 9) was seen in serum LDH, D-dimer, hs-CRP, IL-6, CTSS, procalcitonin, respiratory rate, and chest radiographic findings. SARS-CoV-2 IgG titer increased significantly in the IVIG arm. There was a statistically significant reduction in mortality in the IVIG group (5 vs. 10).

**Conclusion:**

IVIG was a safe and effective adjunctive therapy to SoC treatment in moderate-to-severe COVID-19 patients needing ventilatory support. Furthermore, studies are required to validate our findings. This trial is registered with CTRI/2021/05/033622.

## 1. Introduction

Since February 2020, the world has been overwhelmed by the SARS-CoV-2 virus outbreak, and several patients suffered interstitial pneumonia and respiratory failure requiring mechanical ventilation, threatening the capability of healthcare systems to handle this amount of critical cases. Severe COVID-19 illness may be more than a cytokine storm, acting with more complex mechanisms involving innate and cellular immune responses [1].

Unfortunately, there are a few validated therapies to prevent or treat the severe acute respiratory distress syndrome (ARDS) caused by this novel virus. Thus, the case fatality rate in patients admitted to ICU is exceptionally high. Therefore, along with maintaining vital functions through supportive treatment, effective therapies for COVID-19 are urgently needed.

Immunoglobulins (Ig) have pleiotropic effects on the inflammatory-immune response, including toxin scavenging, microbial phagocytosis, anti-inflammatory effects, and antiapoptotic actions on immune cells. Immunoglobulin may have a role in the early phases of COVID-19 by reducing the viral burden and scavenging or downregulating the production of high inflammatory mediators. In the late phases, especially in ICU patients with secondary bacterial infections, IVIG may have an essential synergic activity in the empowerment of antibiotic efficacy and in supporting overt immune dysfunction [2].

Although the multifaceted immunomodulant properties of IVIG could benefit COVID-19 patients, clinical data supporting adjunctive therapy with IVIG in these patients are few and limited only to the use of standard polyclonal IVIG [3–5]. The crucial role of endogenous immunoglobulins in host response and the robust experience in immunocompromised and septic patients make adjunctive therapy with IVIG attractive in COVID-19, particularly in hospitalized patients with severe respiratory failure. A structured consensus identified the rationale for the use of IVIG therapy in these patients with immune paralysis for preventing secondary infections and in patients with septic shock caused by nosocomial infections [6].

At Lok Nayak Hospital, New Delhi, we conducted a study on moderate-to-severe hospitalized COVID-19-positive cases needing ventilatory support. The study's primary objective was to study the efficacy of intravenous immunoglobulin (IVIG) drug therapy on moderate-to-severe COVID-19 patients. The second objective was to evaluate the safety of IVIG when used in moderate-to-severe hospitalized COVID-19 patients.

## 2. Patients and Methods

### 2.1. Study Design and Population

This was a single-center, randomized, open-label, parallel-group, comparative trial study conducted at Lok Nayak Hospital, New Delhi, to evaluate the efficacy and safety of intravenous immunoglobulin (IVIG) in hospitalized patients with COVID-19 between mid-May 2021 and October 2021.

59 moderate-to-severe laboratory-confirmed reverse transcriptase-polymerase chain reaction (RT-PCR) COVID-19-positive subjects were recruited as per the Ministry of Health and Family Welfare (MoHFW) criteria, which defined moderate illness as an adolescent or adult with the presence of clinical features of dyspnea and/or hypoxia, fever, and cough, including SpO2 ≤94% (range: 90–94%) on room air and respiratory rate more or equal to 24 breaths per minutes [7].

Similarly, severe illness was defined as an adolescent or adult with clinical signs of severe pneumonia plus one of the following: respiratory rate >30 breaths/min, severe respiratory distress, or SpO2 <90% on room air [7].

Clinical data were collected using a structured proforma and then subjected to appropriate statistical analysis. A written informed consent was obtained from all the study participants after explaining the study procedure and the benefits and risks of participation. In severe COVID-19 patients, written consent was taken from their legal guardians. Privacy and confidentiality of data were maintained.

### 2.2. Inclusion and Exclusion Criteria

Inclusion criteria are as follows:

1. Laboratory-confirmed (RT-PCR) COVID-19-positive subjects of moderate-to-severe illness category as per the abovementioned criteria were included.
2. Age 18 to 65 years

Exclusion criteria are as follows:

1. Subjects with known hypersensitivity to immunoglobulin,
2. History of deep vein thrombosis (DVT),
3. Pulmonary embolism (PE),
4. Thromboembolic stroke or other thrombotic events,
5. Pregnant females, and
6. Active participants in another research treatment study are to be excluded.

### 2.3. Sample Size Estimation

To the best of our knowledge, it was a first-of-its-kind pilot study; hence, a convenience sample of 40 patients (20 patients at least per group) was taken. Adjusting for 20% dropouts, approximately 24 patients per group, at least 48 patients were to be enrolled in the study.

### 2.4. Methodology

**Figure 1:**
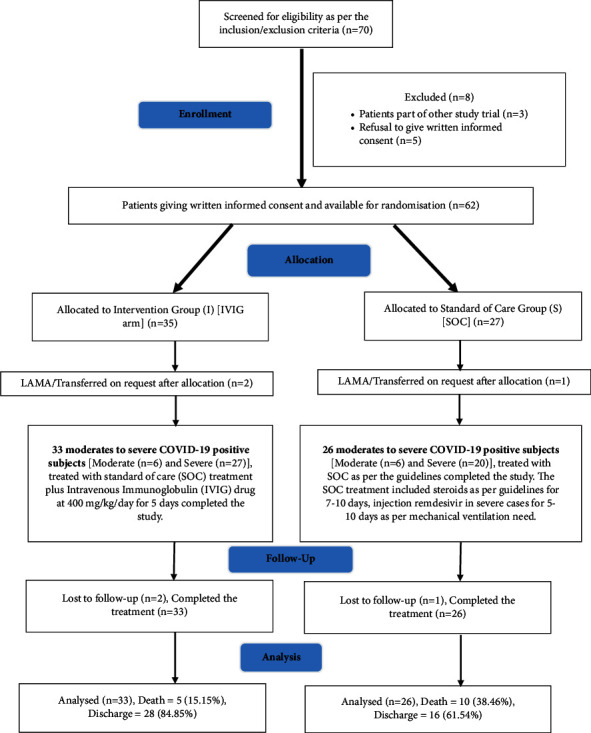
Study flow diagram of randomized controlled trial.

Seventy eligible patients were screened for the study as per the inclusion and exclusion criteria, as shown in Figure 1.

Eight patients were excluded due to involvement in other studies (*n* = 3) or refusal to give written informed consent (*n* = 5). Sixty-two patients were available for the allocation to the groups.

Sixty-two available study subjects willing to provide written informed consent were divided into two treatment arms: the first treatment arm (IVIG group) with 35 moderate-to-severe COVID-19-positive subjects treated with standard-of-care (SoC) treatment plus intravenous immunoglobulin (IVIG) drug at 400 mg/kg/day for five days and the second treatment arm (SoC group) with 27 moderate-to-severe COVID-19-positive subjects treated with SoC as per the guidelines, as shown in Fig. 1. The IVIG was initiated within 48 hours of hospital admission and given as a slow infusion (initially started with 0.5 mL/kg/h and gradually titrated to a maximum of 2 mL/kg/h) along with adequate hydration.

Subjects underwent simple randomization via open labeling, where each study subject was allocated to IVIG (intervention) and S-SoC groups using computer-generated codes. Patients were allocated to different groups depending on odd or even numbers through a simple random number generation in the Excel sheet.

The SoC treatment included steroids per guidelines for 5–10 days and remdesivir in severe cases for 5–10 days as per the need for mechanical ventilation. IV methylprednisolone 0.5 to 1 mg/kg and 1 to 2 mg/kg IV in two divided doses were given for 5–10 days in moderate and severe patients, respectively [7, 8]. A per-protocol analysis was done wherein all the allocated subjects were interviewed and underwent detailed clinical history, including demographic details; history of COVID-19 vaccination; pre-existing medical conditions, such as hypertension, diabetes, tuberculosis, heart or liver, or kidney disease or any allergic reaction; and examination as per a structured proforma. For severe patients, a relevant history was obtained from their legal guardian. National Early Warning Score 2 (NEWS2) and routine vitals, including BP (blood pressure), respiratory rate, pulse rate, random blood sugar, and oxygen saturation via pulse oximeter, were measured on the admission of every patient. These vitals were monitored subsequently throughout the hospital stay for every patient.

Furthermore, study subjects underwent a baseline routine laboratory analysis including serum inflammatory markers (hs-CRP, D-dimer, ferritin, and IL-6), IgG titer (the presence of antibodies against SARS-CoV-2), bedside chest radiograph, and an HRCT (high-resolution CT) scan of the chest, and a CTSS was calculated based on a defined radiological criterion [9].

NEWS2 was calculated using respiratory rate, oxygen saturation, systolic blood pressure, heart rate, level of consciousness, and temperature. The score was then calculated, and two points were added in case of supplemental oxygen need [10].

IgG titers (*μ*g/ml) were examined by enzyme-linked immunosorbent assay (ELISA) ErbaLisa® COVID-19 IgG semiquantitative kit. Repeat NEWS2, inflammatory markers, IgG titers, CTSS, and chest radiograph findings were noted on Day 9^th^ of hospitalization, which were then compared between the IVIG and SoC treatment groups.

As per Government of India guidelines, patients were discharged after ten days of symptom onset if symptoms resolved within three days and saturation above 95% was maintained for the next four days (without oxygen support) [11].

Thirty-three patients in the IVIG study group and 26 in the SoC group completed the entire study and were analyzed concerning the outcome parameters. Two patients in the IVIG group and one in the SoC group dropped out midway through the study due to a patient/attendant requesting transfer or LAMA (leave against medical advice).

### 2.5. Outcome Variables

#### 2.5.1. The Primary Outcome

The primary outcome was to study the efficacy of intravenous immunoglobulin (IVIG) drug therapy on moderate-to-severe COVID-19 patients in terms of clinical improvement as measured by improvement in the NEWS2 at the end of treatment.

#### 2.5.2. The Secondary Outcome

The secondary outcome was to evaluate the safety of IVIG when used in moderate-to-severe COVID-19 patients and to study the change in oxygen saturation by pulse oximetry, all-cause mortality until the duration of hospital stay, average number of days of hospitalization, change in inflammatory markers, IgG titer, CTSS score, and radiological findings between the IVIG and SoC treatment groups.

### 2.6. Statistical Method

The data were compiled and analyzed using MS Excel (R) Office 365, GraphPad Prism 8.4.2, and SPSS version 25. Descriptive statistics were presented as proportions/percentages for categorical variables and mean and standard deviation for continuous data variables. Fisher's exact test/Chi-square test was used to compare proportions (categorical variables). Continuous variables were analyzed using the Mann–Whitney test/Student's *t*-test (independent group/unpaired data) and Wilcoxon sign-rank test/paired *t*-test (for paired data) based on the normality of the data. A *P* value of ≤0.05 was considered significant.

## 3. Results

### 3.1. Demographic and Clinical Parameters

A total of 59 subjects completed the study. “The average age and gender distribution across the two groups were similar with no significant difference statistically, as shown in Table 1.” Both the groups were similar in comorbidity profile, clinical severity scoring, CT-based severity scoring, and NEWS2 at the baseline (Day 1). Apart from pulse rate and SpO2 levels at baseline, all other disease-related parameters were similar across the two groups. Thus, the demography parameters showed no significant difference between the two groups.

### 3.2. Laboratory Parameters

**Figure 2:**
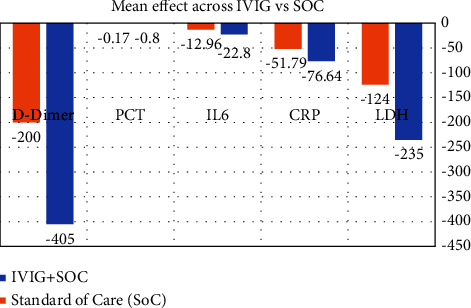
Showing mean of difference assessment (absolute effect) between the IVIG and SoC groups.

Laboratory profiles of the patients comparing Day 1 and Day 9 values have been tabulated in Table 2. It was seen that the IVIG group had a significant change in the P/L ratio, neutrophil, and lymphocyte proportion as compared to baseline. Similar trends were not seen in the SoC group. The rest of the hematological and biochemical parameters were comparable between Day 1 and Day 9. The serum inflammatory marker (LDH, hs-CRP, IL-6, procalcitonin, and D-dimer) levels also significantly improved in the IVIG and SoC groups. However, the extent of improvement, as suggested by the mean of difference assessment (absolute effect), as shown in Figure 2, was higher in the IVIG group.

Serum D-dimer levels were seen to fall by 405 (95% CI: −727.18 to −82.81) ng/ml in the IVIG group vs 200 (95% CI: −685.13 to 283.95) ng/ml in the SoC group. Similarly, serum LDH levels reduced by 235 (95% CI: −336.24 to −133.89) U/L compared to 124 (95% CI: −243.76 to −4.23) U/L in the IVIG group. The change in the level of serum IL-6, D-dimer levels, and procalcitonin was not significant in the SoC group but was significant in the IVIG group.

It was further seen that the serum IgG levels on Day 9 increased significantly compared to baseline in the IVIG group (6.58 vs 3.59 *μ*g/ml at baseline). A slight increase was seen in the SoC group (4.88 vs 4.53 *μ*g/ml), but the absolute effect was much higher in the IVIG group [2.99 (95% CI: 2.05 to 3.92) vs 0.350 (95% CI: −0.61 to 1.31)] (*P* value <0.0001).

### 3.3. Chest Radiograph Profile

The findings suggested that there was a significant improvement in the chest radiograph findings in the IVIG group on Day 9 in terms of the proportion of patients with GGO (87.88% vs. 100% at baseline), central (24% vs. 48% at baseline) and peripheral lung infiltration (87% vs. 100% at baseline), basal area involvement (78% vs. 96% at follow-up), and pleural effusion (9% vs. 39% at baseline) on Day 9 compared to that at the start of therapy initiation. No such trends were seen in the SoC group (Table 3).

### 3.4. Outcome Parameters

The outcome profiles of the patients have been tabulated in Table 4. It was observed that a significant improvement was seen in the respiratory rate in the IVIG group (19.86 vs 25.33 per minute at baseline) compared to the SoC group. The absolute change in the respiratory rate was significant in the IVIG group: −5.47 (95% CI: −7.35 to −3.58). The SoC group had no significant change in the respiratory rate on Day 9 compared to Day 1. The respiratory rate was almost similar, with no clinically noticeable change.

**Figure 3:**
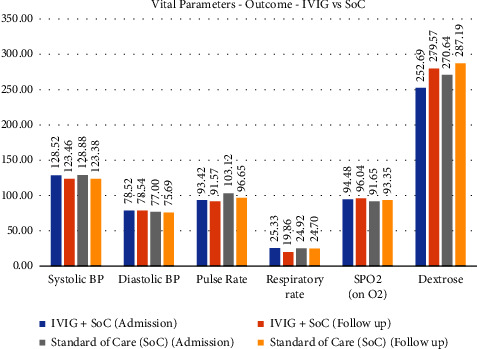
Showing vital parameters outcome in the IVIG and SoC group.

SpO2 improvement was seen across both the groups, with the extent of improvement comparable across both the groups [(95% CI: 1.70 in SoC vs 1.56 in the IVIG group)]. The trend was significant for the SoC group and was very close to significance in the IVIG group. Vital parameter change is shown in Figure 3.

Similarly, the IVIG group significantly improved the CTSS (17.97 on Day 9 vs 21.67 on Day 1). A slight decrease in the CTSS was seen in the SoC group, but the trend was insignificant (−0.540 in the SoC group vs −3.70 in the IVIG group).

Additionally, the NEWS2 declined significantly across both groups. The extent of improvement was the same across both groups (−1 in IVIG vs −0.96 in SoC). The mean of effect between Day 1 and Day 9 showed no significant difference between the groups [−1.00 (−1.88 to −0.11) in IVIG vs. −0.96 (−1.54 to −0.37) in SoC].

The mortality was significantly lower in the IVIG group compared to the SoC group (15.1% vs 38.4%). The average duration of hospital stay was significantly higher in the SoC group (25 days) compared to the IVIG group (18 days). This difference was significant statistically (*P* < 0.0001).

## 4. Discussion

The lack of an effective antiviral agent and adequate vaccination coverage during the Delta wave (B.1.617.2) of COVID-19 in India complicated the situation and called for further intensification in the research. As per an Indian study, the Delta COVID-19 wave had been associated with a higher case fatality rate, affecting younger individuals and more rampant hospitalization, subsequently overburdening healthcare facilities [12]. In parallel with developing new agents, studying the efficacy of existing therapeutic options with an acceptable safety profile was prudent. The intravenous immunoglobulin (IVIG) administration represented such an example [13]. In the COVID-19-related multisystemic inflammatory syndrome in children and adolescents, the Ministry of Health, Government of India, has considered intravenous immunoglobulins as the first line of treatment [14]. Hence, this randomized, open-label parallel-group study was conducted in Lok Nayak Hospital, New Delhi, from mid-May to October 2021 to evaluate the efficacy and safety of intravenous immunoglobulin (IVIG) in moderate-to-severe hospitalized COVID-19 patients.

The present study enrolled a population of moderate-to-severe hospitalized COVID-19-positive cases needing ventilatory support for evaluating adjuvant therapy with IVIG and the standard of care (SoC). Studies have found that early administration of IVIG (within three days) during the COVID-19 infection has improved patient recovery and survival rates [15–17]. IVIG was initiated within 48 hours after hospitalization in the present study. Evidence also suggests that high-dose IVIG (0.3–0.5 g per kg weight for five doses) with combined corticosteroids could have a better outcome and may be considered valid and safe immunotherapy in COVID-19 patients [18]. Comparing the baseline demographic and clinical characteristics of the patients, it was, however, seen that the pulse rate was higher and SpO2 levels were lower in the SoC group, which might suggest that more clinically severe patients were a part of the SoC group that could imply the overall outcomes, but given the fact that CTSS and clinical severity grade-based distribution was similar across the two groups, this difference was unlikely to have a significant clinical impact on outcomes.

### 4.1. Anti-Inflammatory or Immunomodulatory Role

The overall effects of adding IVIG in our study could be attributed to its anti-inflammatory and immunomodulatory properties. There was a statistically significant reduction in the serum inflammatory marker levels (LDH, hs-CRP, IL-6, procalcitonin, and D-dimer) in the IVIG group, as shown in Figure 2. More strikingly, the absolute fall in the serum D-dimer [405 (95% CI: −727.18 to −82.81) ng/mL vs. 200 (95% CI: −685.13 to 283.95) ng/mL] and serum LDH levels [235 (95% CI: −336.24 to −133.89) U/L vs. 124 (95% CI: −243.76 to −4.23) U/L] was seen more in the IVIG group than SoC group on Day 9. Similarly, the extent of improvement in the chest radiography findings, as shown in Table 3, on Day 9 was significant in the IVIG group in terms of the proportion of patients with GGO, central and peripheral lung infiltration, basal area involvement, and pleural effusion as compared to Day 1. Moreover, the CTSS fell by −3.70 (95% CI: −5.22 to −2.17) in the IVIG group vs −0.540 (95% CI: −2.46 to 1.38) in the SoC group. The absolute change in the respiratory rate was significant in the IVIG group as compared to the SoC group on Day 9 compared to Day 1 (Table 4). This reflected the additional anti-inflammatory role of IVIG compared to the SoC, as seen on Day 9. A retrospective cohort study also reported a significant decrease in serum CRP and ferritin levels on Day 6 following IVIG therapy for five days [19]. Similar effects have also been found in other studies [20]. Literature shows that these immunomodulatory effects of IVIG come through the blockage of intact Fc*γ* receptors on the immune cells, downregulating the inflammatory cytokines like IL-6 and TNF-alpha and inhibiting the complement cascade [21–23].

IVIG comprises human immunoglobulins, predominantly IgG, pooled from thousands of healthy plasma donors who have recovered after viral infections. It contains neutralizing antibodies against microbial infections in improved patients from various infections. In addition, neutralizing antibodies, autoantibodies, and natural antibodies are found in prepared IVIG [24]. Our study found that SARS-CoV-2-specific IgG titer was significantly higher in the IVIG group on Day 9 than SoC. Díez, J in their study supports the presence of anti-SARS-CoV-2 cross-reacting antibodies in the IVIG preparations, where a cross-reactivity of IVIG products with SARS-CoV-2, SARS-CoV, and MERS-CoV was found using ELISA test on the available IVIG preparations [25]. Also, IVIG preparations have been shown to contain antibody reactivity against the SARS-CoV-2 S1 protein [25]. Moreover, in IVIG preparations created during the pandemic year, Schwaiger, J in their study showed a substantial rise in the concentration of specific neutralizing antibodies against SARS-CoV-2 [26].

### 4.2. Effect on Clinical Profile

A study conducted by Ina Kostakis and colleagues supports the national and international recommendations for the use of NEWS or NEWS2 for the assessment of acute illness severity in hospitalized patients with COVID-19 [27–30]. A study by Marius-Myrstad and colleagues found that the NEWS2 at hospitalization predicted severe disease and in-hospital mortality and was superior to other clinical risk scores like qSOFA, SIRS criteria, and CRB-65 score in COVID-19 patients [31]. Similarly, another study found that the NEWS was more sensitive in predicting in-hospital mortality, early bacterial infection, and ICU admission than SIRS and qSOFA [32]. Hence, assessing the change in the NEWS2 was used as the primary outcome of our study.

The clinical improvement measured by improvement in the National Early Warning Score 2 (NEWS2) at the end of treatment did not show a statistically significant difference, as the extent of improvement was the same across both groups (−1 in IVIG vs −0.96 in SoC). However, a study by Reynaga E and colleagues showed a rapid improvement in NEWS, reaching a minimum value by 14 days post-IVIG administration in five subjects with a medium risk of ARDS and a mid-to-high NEWS [33]. The effect of the IVIG on the NEWS2 could have been more substantial if a larger sample-sized study population had been assessed.

Moreover, in our study, there was better control of respiratory rate in the IVIG group as shown by the absolute change in the respiratory rate in the IVIG group as −5.47 (95% CI: −7.35 to −3.58) compared to the SoC group on Day 9 as compared to Day 1. Similarly, the CTSS score fell significantly by −3.70 (95% CI: −5.22 to −2.17) in the IVIG group vs −0.540 (95% CI: −2.46 to 1.38) in the SoC group. A case series of five patients who received IVIG showed improved pulmonary involvement after five days [23]. The study by Zhou et al. documented a reduction in pulmonary shadows in 36 cases out of 40 who received IVIG and steroids [34].

The SpO2 improvement was seen across both the groups, and the extent of improvement was comparable across both the groups (95% CI: 1.70 in SoC vs 1.56 in the IVIG group). This again highlights that IVIG is essential in combating the disease process by having an immunomodulatory or anti-inflammatory effect in COVID-19 patients. Once the multifaceted effects of IVIG alter the pathology, one could see the more pronounced effect in the overall outcome in terms of duration of hospital stay and mortality. An improvement in SpO2 values after IVIG treatment in COVID-19 patients has also been validated in multiple other studies [16, 23, 34, 35]. Similarly, in another case study report, the oxygen saturation level was above 96% after six days of IVIG treatment without oxygen support [17].

### 4.3. Effect on Outcome

The number of hospitalization days was significantly lower (18 days vs 25 days) in the IVIG arm; similarly, the number of deaths (15% vs 38%) in the IVIG study arm was considerably less than in the SoC arm. Conversely, the number of subjects discharged at the end of our study was 84.85% in the IVIG group compared to the standard-of-care group (61.54%). Similar results were reported by a randomized, open-label study by Raman et al. in moderate COVID-19 patients [36]. However, they reported mild-to-moderate adverse reactions following IVIG infusion, which were not encountered in our study. Similarly, a retrospective study from China involving 58 severe and critically ill COVID-19 patients showed that early institution of IVIG within 48 h of admission to ICU was associated with a reduction in mechanical ventilation use, shortened ICU duration, and ICU and hospital stay, and improved 28-day survival [15].

Furthermore, it was found that severe ARDS was the primary cause of mortality among the study subjects in our study. Few other studies have also found adverse effects like thromboembolism [37, 38] and acute kidney injury [39] with IVIG, which could have led to increased mortality. Keeping a slower IVIG infusion rate, along with hydration and enoxaparin use as per MoHFW criteria, might have avoided these adverse effects in our study [7, 8]. Moreover, no secondary infections were seen in the IVIG group, as serum procalcitonin and TLC levels were within the normal range. This could also point toward the anti-inflammatory and anti-infectious nature of the IVIG. Moreover, in the present study, IVIG was used with other recommended therapeutic options available as per the norms, such as methylprednisolone and remdesivir, based on the severity of COVID-19 infection. The overall effect of these therapies could have resulted in beneficial outcome.

On the other hand, a retrospective, multicenter cohort study assessing the outcome of IVIG in critically ill COVID-19 patients found no remarkable differences in hospitalization, ICU length of stay, duration of mechanical ventilation, and even mortality rate [40]. A multicenter, double-blinded, placebo-controlled, and phase 3 trial involving moderate-to-severe COVID-19 ARDS showed that IVIG did not improve clinical outcomes at Day 28 and showed an insignificant increase in adverse events [41]. Yet, another randomized controlled trial involving 84 severe COVID-19 patients IVG (400 mg/kg, IV, daily for three days) was given to 52, and 32 were in the control group. The study did not support the beneficial effects of IVIG in combination with hydroxychloroquine and lopinavir/ritonavir SARS-CoV-2 patients, as improvement in the mortality rate, radiographic changes, and the need for mechanical ventilation was not evident; however, a positive relationship was found between early IVIG initiation and the length of the ICU and hospital stay [42]. This again highlights the importance of using IVIG earlier during COVID-19 infection, as seen in our study.

It is evident that IVIG use in COVID-19 has been associated with a mixed response [43, 44]; however, our study found the early institution of IVIG to be a safe and effective adjunctive therapy in moderate-to-severe COVID-19 infection. Furthermore, as a pilot study, our study provides valuable inputs on assessing the role of adjunctive IVIG over SoC on the NEWS2 among hospitalized moderate-to-severe COVID-19 patients. However, much larger trials are needed to validate the study's findings. Given the immune system redundancies, IVIG appears to offer global, multifaceted mechanisms of immunomodulation to counteract the immune dysregulation (i.e., “cytokine storm”) seen in severe COVID-19 infection.

### 4.4. Limitations of the Study

The main limitation of our study was that we could not rule out the possible advantages of IVIG monotherapy in treating COVID-19 since ethical concerns prevented us from including a control group without any treatment. Additionally, the sample size of our study was relatively small, which may limit the generalizability of our findings. Our study was conducted during the peak of Delta wave, and hence, more patients were enrolled to achieve a greater sample size initially. However, as the study progressed, the Delta wave subsided suddenly with nearly no inflow of patients resulting in a disproportionate allocation of patients among the treatment groups, potentially impacting the balance between the subgroups. Moreover, had we used block randomization, a discrepancy in the study subjects between the groups could have been avoided. Additionally, our trial was conducted in an open-label manner, lacking blinding, which may have influenced the interpretation of the results. Moreover, the study could have been more meaningful if we had included a follow-up of the patients to assess the long-term effects of IVIG. Furthermore, the cost and limited accessibility of IVIG therapy in resource-poor settings raise concerns about the feasibility of implementing our findings in clinical practice. Finally, we recognize the need for more extensive clinical studies to validate our findings and provide more robust evidence regarding the efficacy and safety of IVIG therapy in treating COVID-19.

## 5. Conclusions

To conclude, IVIG was found to be a safe and effective therapy adjunctive to SoC treatment in moderate-to-severe hospitalized COVID-19 patients needing ventilatory support. The study consolidates the previous findings of the promising role of the addition of IVIG to the standard of care. The clinical difference was appreciated through the anti-inflammatory effects of IVIG in most of the severity outcomes as well as the outcome profile with an overall improvement in the reduction of NEWS2, CT severity score, inflammatory markers, duration of hospital stay, deaths, and improvement in respiratory rate, SpO2 values, and IgG titers from the baseline. Furthermore, large-scale studies are needed to validate our findings.

## Figures and Tables

**Table 1:** Baseline (Day 1) demographic and clinical profile.

Parameters	IVIG + SoC group		Standard of care (SoC)		*P* value
Number	33		26		
Age (years)					
Mean	56.94		59.73		0.1770
SD	8.69		6.44		
Range	33–73		48–72		
Gender					
Male	22	66.67%	13	50%	0.1729
Female	11	33.33%	13	50%	
Comorbidity profile					
Hypertension	25	75.76%	21	81%	0.6079
T2 diabetes mellitus	18	54.55%	16	62%	0.5425
Obesity	4	12.12%	1	4%	0.2292
Bronchial asthma	1	3.03%	1	4%	0.8409
Chronic obstructive pulmonary disease	1	3.03%	1	4%	0.8409
Hypothyroidism	5	15.15%	4	15%	0.9045
Coronary artery disease	6	18.18%	5	19%	0.8113
Dilated cardiomyopathy	1	3.03%	0	0%	0.3748
Old pulmonary tuberculosis	4	12.12%	2	8%	0.6084
Baseline vital parameters (day 1)					
Systolic BP (mm·Hg)	128.52 ± 17.21		128.88 ± 17.05		0.9185
Diastolic BP (mm·Hg)	78.52 ± 10.37		77.00 ± 9.31		0.7839
Pulse rate (beats/min)	93.42 ± 10.47		103.12 ± 16.43		**0.0078**
Respiratory rate (rate/min)	25.33 ± 3.89		24.92 ± 2.06		0.6033
SPO2 (on O2 support)	94.48 ± 3.79		91.65 ± 3.25		**0.0037**
Random blood sugar (mg/dL)	252.69 ± 97.43		270.64 ± 99.69		0.5317
Clinical severity					
Moderate	6	18.18%	6	23%	0.6505
Severe	27	81.82%	20	77%	
NEWS2 at admission (Day 1)					
Mean	6.67		6.92		0.3907
SD	1.08		1.13		
Range	4–8		3–9		
CTSS score (Day 1)					
Mean	21.67		21.23		0.5141
SD	2.25		2.90		
Range	17–25		13–25		

Bold values denote significant *P*-values (≤0.05).

**Table 2:** Laboratory parameters.

Lab parameter	IVIG + SoC (Day 1)	IVIG + SoC (Day 9)	*P* value	SoC (Day 1)	SoC (Day 9)	*P* value
Number	33	33		26	26	
Hb (12-15.5 g/dL)	12.08 ± 1.83	12.30 ± 1.50	0.5957	12.55 ± 2.04	12.28 ± 1.39	0.681
TLC (4000–11000 cells/mm^3^)	10736 ± 4900	10443 ± 4094	0.7929	13666 ± 5330	13798 ± 6087	0.925
Polymorphocytes (60–75%)	87 ± 5	79 ± 8	**<0.001**	89 ± 10	87 ± 9	0.396
Lymphocytes (2–6%)	11 ± 5	18 ± 8	**0.0001**	9 ± 9	11 ± 7	0.317
P/L ratio	1.82 ± 0.82	2.31 ± 0.93	**0.0266**	2.20 ± 0.86	2.23 ± 1.07	0.900
Urea (19–43 mg/dL)	38.09 ± 19.22	36.23 ± 14	0.6558	52.15 ± 26.71	42.50 ± 24.96	0.658
Creatinine (0.66–1.26 mg/dL)	0.71 ± 0.22	0.73 ± 0.24	0.5187	0.74 ± 0.35	0.67 ± 0.33	0.821
Na (137–145 mmol/L)	138.76 ± 3.67	137.71 ± 3.58	0.8330	138.58 ± 3.50	137.08 ± 7.14	0.661
K (3.5–5.1 mmol/L)	4.52 ± 0.51	4.59 ± 0.42	0.7015	4.60 ± 0.41	4.40 ± 0.46	0.325
T. Bilirubin (1.3 mg/dL)	0.84 ± 0.34	0.76 ± 0.25	0.2803	0.81 ± 0.43	0.76 ± 0.29	0.305
ALT (5–50 U/L)	79.76 ± 157.25	61.52 ± 79.15	0.2189	63.35 ± 53.91	111.12 ± 181.33	0.641
AST (15–45 U/L)	57.88 ± 56.84	45.19 ± 35.65	0.3911	51.50 ± 26.70	61.92 ± 53.63	0.705
ALP (38–125 U/L)	129.41 ± 50.47	119.06 ± 45.83	0.5019	102.81 ± 50.57	91.31 ± 41.90	0.611
S. Lactate (0.5–1.5 mmol/L)	2.17 ± 0.55	1.92 ± 0.99	0.2094	2.43 ± 0.81	2.33 ± 0.59	0.613
PCT (low risk: <0.5, high risk: >2 ng/mL)	0.94 ± 1.20	0.14 ± 0.20	**0.0003**	0.91 ± 0.79	0.74 ± 0.48	0.084

*Inflammatory markers (serum)*						
LDH (125–146 U/L)	643.52 ± 254.56	408.45 ± 140.86	**<0.001**	728.38 ± 229.43	604.08 ± 200.94	**0.042**
CRP (0.0–0.5 mg/dL)	96.17 ± 52.19	19.53 ± 18.83	**<0.001**	118.62 ± 100.92	66.83 ± 59.39	**0.028**
IL6 (<6 pg/mL)	42.41 ± 31.90	19.52 ± 47.65	**0.0251**	73.64 ± 94.46	60.68 ± 101.69	0.636
S. Ferritin (30–400 ng/mL)	647.80 ± 352.44	614.66 ± 392.81	0.5501	659.81 ± 320.37	638.16 ± 318.71	0.687
INR (0.9–1.1)	1.07 ± 0.19	1.01 ± 0.10	0.1134	1.09 ± 0.11	1.08 ± 0.26	0.809
D-dimer (<500 ng/mL)	1059.31 ± 667.47	654.87 ± 643.54	**0.0146**	1353.55 ± 961.24	1152.96 ± 767.55	0.409

Bold values denote significant *P*-values (≤0.05).

**Table 3:** Chest radiograph findings and their comparison between groups.

Chest x-ray parameters	IVIG + SoC (Day 1)		IVIG + SoC (Day 9)		*P* value	SoC (Day 1)		SoC (Day 9)		*P* value
Number	33					26				
Ground glass opacities (GGO)	33	100.00%	29	87.88%	**0.0406**	26	100%	26	100%	—
Consolidation	30	90.91%	29	87.88%	0.6916	24	92%	24	92%	—
Central	16	48.48%	8	24.24%	**0.0422**	20	77%	18	69%	0.5199
Peripheral	33	100.00%	29	87.88%	**0.0406**	26	100%	25	96%	0.3076
Apical	15	45.45%	9	27.27%	0.1276	18	69%	14	54%	0.2710
Basal	32	96.97%	26	78.79%	**0.0247**	26	100%	26	100%	—
Hilar lymphadenopathy	9	27.27%	8	24.24%	0.7800	5	19%	5	19%	—
Pleural effusion	13	39.39%	3	9.09%	**0.0044**	12	46%	7	27%	0.1588

Bold values denote significant *P*-values (≤0.05).

**Table 4:** The vital parameter-related outcome, CTSS, and NEWS2.

Parameters	IVIG + SoC (mean ± SD) Day 1	IVIG + SoC (Day 9)	*P* value	Standard of care (SoC) (Day 1)	Standard of care (SoC) (Day 9)	*P* value
Number	33			26		
Systolic BP (mm Hg)	128.52 ± 17.21	123.46 ± 12.15	0.1725	128.88 ± 17.05	123.38 ± 14.69	0.206
Diastolic BP (mm Hg)	78.52 ± 10.37	78.54 ± 10.10	0.8331	77.00 ± 9.31	75.69 ± 12.46	0.519
Pulse rate	93.42 ± 10.47	91.57 ± 9.86	0.5027	103.12 ± 16.43	96.65 ± 16.97	0.228
Respiratory rate	25.33 ± 3.89	19.86 ± 3.77	**<0.0001**	24.92 ± 2.06	24.70 ± 3.06	0.982
SPO2 (on O2 support)	94.48 ± 3.79	96.04 ± 2.78	0.0611	91.65 ± 3.25	93.35 ± 2.64	**0.043**
Random blood sugar (mg/dL)	252.69 ± 97.43	279.57 ± 125.37	0.6144	270.64 ± 99.69	287.19 ± 153.30	0.701

*CTSS*						
Mean	21.67	17.97	**<0.0001**	21.23	20.69	0.5754
SD	2.25	3.77		2.90	3.93	

*NEWS2*						
Mean	6.67	5.67	**0.0277**	6.92	5.96	**0.0019**
SD	1.08	2.31		1.13	0.97	

Bold values denote significant *P*-values (≤0.05).

## Data Availability

The data used to support the findings of this study are available from the corresponding author upon request.

## Additional Points

*Clinical Trail Registration*. The study was registered in the Clinical Trials Registry of India (CTRI) with reference no: CTRI/2021/05/033622 (Registered on 14/05/2021).
